# Multiplex PCR analysis of clusters of unexplained viral respiratory tract infection in Cambodia

**DOI:** 10.1186/s12985-014-0224-x

**Published:** 2014-12-17

**Authors:** Nary Ly, Rafal Tokarz, Nischay Mishra, Stephen Sameroff, Komal Jain, Agus Rachmat, Ung Sam An, Steven Newell, Dustin J Harrison, W Ian Lipkin

**Affiliations:** U.S. Naval Medical Research Unit-2 Detachment, Phnom Penh, Cambodia; Center for Infection and Immunity, Mailman School of Public Health, Columbia University, New York City, USA; National Institute of Public Health, Cambodian Ministry of Health, Phnom Penh, Cambodia

**Keywords:** Enterovirus 68, Respiratory disease, RSV

## Abstract

**Background:**

Fevers of unknown origin constitute a substantial disease burden in Southeast Asia. In majority of the cases, the cause of acute febrile illness is not identified.

**Methods:**

We used MassTag PCR, a multiplex assay platform, to test for the presence of 15 viral respiratory agents from 85 patients with unexplained respiratory illness representing six disease clusters that occurred in Cambodia between 2009 and 2012.

**Results:**

We detected a virus in 37 (44%) of the cases. Human rhinovirus, the virus detected most frequently, was found in both children and adults. The viruses most frequently detected in children and adults, respectively, were respiratory syncytial virus and enterovirus 68. Sequence analysis indicated that two distinct clades of enterovirus 68 were circulating during this time period.

**Conclusions:**

This is the first report of enterovirus 68 in Cambodia and contributes to the appreciation of this virus as an important respiratory pathogen.

## Introduction

Acute respiratory infections (ARI) are among the leading causes of morbidity and mortality in both developed and developing nations [1-3]. In Cambodia, pneumonia is the third highest cause of death in children under five [4,5]. In December 2006, a passive hospital-based surveillance study was initiated to identify the causes of acute undifferentiated fever in patients seeking healthcare in Cambodia. Between 2006 and 2009, 9,997 patients from peri-urban and rural Cambodian health centers were enrolled in the study. The most frequent diagnoses included influenza, dengue, malaria and leptospirosis [6]. No diagnosis was obtained in 62% of cases [6]. In a separate analysis using samples from December 2006 to December 2008, 4,233 patients with respiratory disease were screened for influenza virus by real-time reverse-transcriptase polymerase chain reaction (rRT–PCR). Of these patients, 1,151 (27.2%) were positive for influenza [7]. In a separate study, Sreng *et al.* analyzed 2,805 patients with influenza-like illness in Cambodia between 2006 and 2008 and showed that only 9.6% tested positive for influenza [8]. To address the high rate of undiagnosed ARI cases in Cambodia, we selected six clusters of previously unexplained respiratory illness from 2009 to 2012 for analysis by MassTag PCR, a high throughput multiplex screening platform. We identified a potential viral pathogen in 44% (37/85) of the samples, confirming the utility of multiplex diagnostic assays in clinical microbiology and providing insights into ARI in Cambodia.

## Results

**Figure 1:**
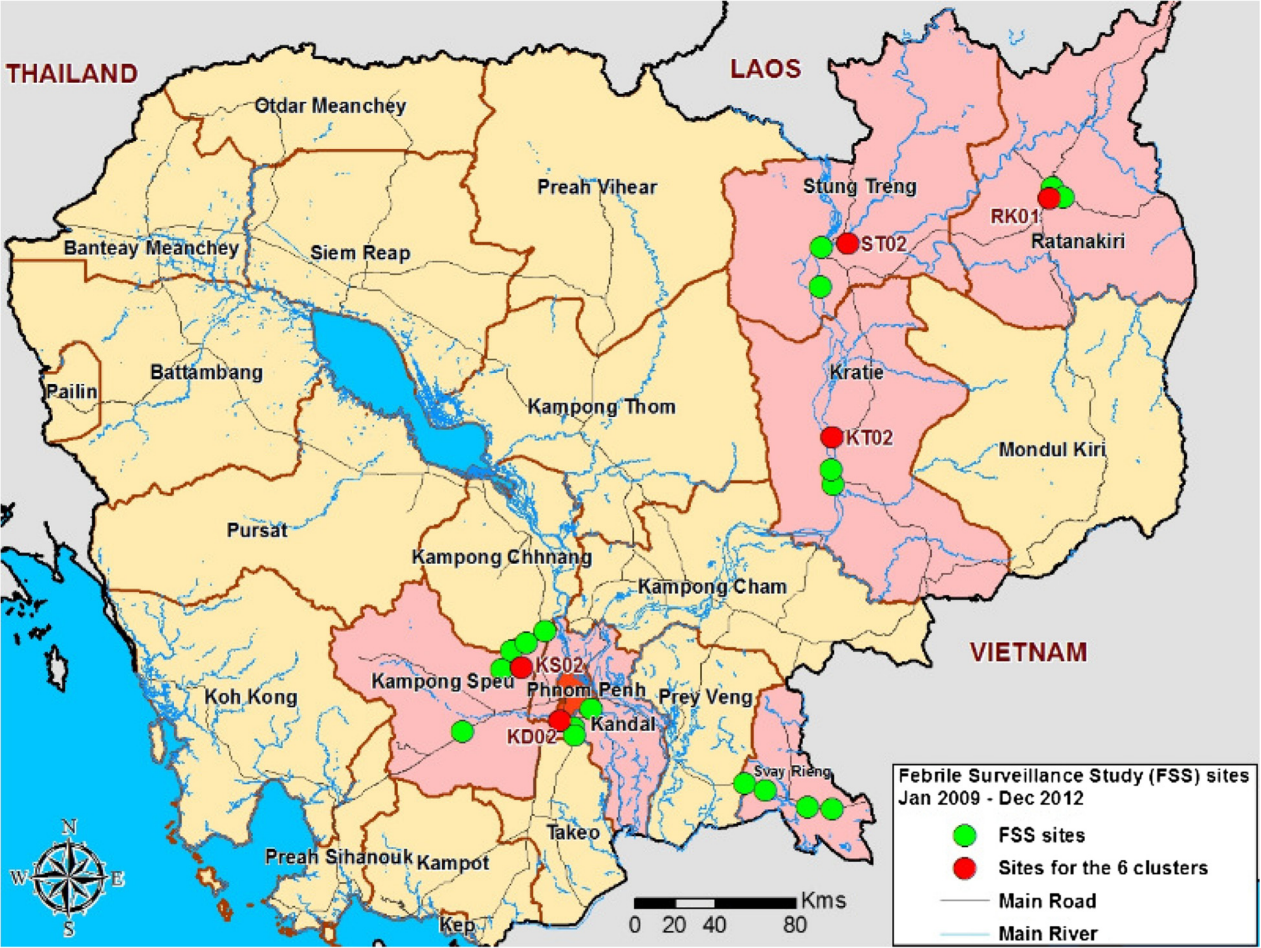
**Map of NAMRU-2PP Febrile Surveillance Study health center sites from January 2009 to December 2012.** The locations of the 6 clusters are indicated in red. Pink represents the 6 provinces where the study was conducted.

**Figure 2:**
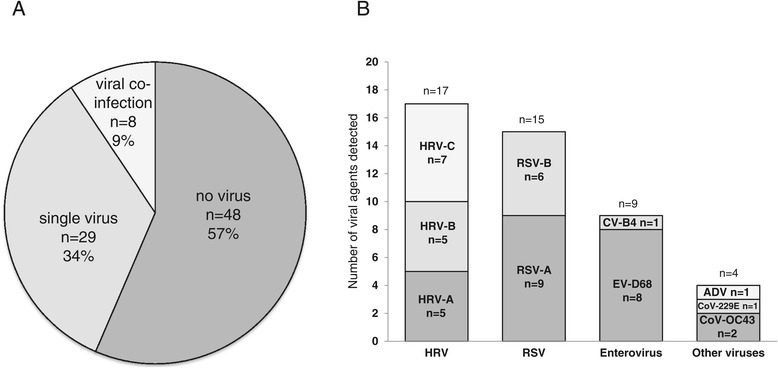
**Summary of agents detected by MassTag PCR. A)** Percentage of viral-positive samples **B)** Distribution of viruses detected in all samples. Each bar represents the number of all detected viruses.

From 2009 to 2012, 17,363 samples were collected from 18 health clinics in six provinces of Cambodia and screened for influenza virus, dengue virus and *Plasmodium* spp (Figure 1). From amongst the samples that tested negative for these agents, we selected 6 clusters representing 85 patients with unexplained acute respiratory illness for further analysis. 88% (75/85) of the patients exhibited influenza-like symptoms (Table 1). The archived specimens from the 6 clusters were screened by MassTag PCR for viral respiratory pathogens [9,10]. At least one virus was detected in 44% (37/85) samples (Figure 2A). Infection with two viruses was detected in 8 samples. Rhinovirus (HRV) was the most frequently detected virus (n = 17), followed by respiratory syncytial virus (RSV; n = 15), and enterovirus (HEV; n = 9) (Figure 2B).

**Table 1:** **Demographics and clinical characteristics of the 6 analyzed clusters**

**Year**	**2009**		**2010**	**2011**	**2012**	
province (site code)	Kandal (KD02)	Kampong Speu (KS02)	Kandal (KD02)	Ratanakiri (RK01)	SteungTreng (ST02)	Kratie (KT02)
enrollment period, (day duration)	24-31 Aug (8 d)	12-16 Oct (7 d)	05-18 Mar (14 d)	25 Jul-12 Sep (50 d)	13-16 Feb (4 d)	4 Apr (1 d)
season	Rainy	Rainy	Dry	Rainy	Dry	Dry
number of cases	20	22	11	11	11	10
median age [range]	36.5 [4–71]	11 [2–60]	26 [6–45]	4 [2–5]	19 [18–49]	23 [2–43]
female/male, n (%)	9 (45)/11 (55)	12 (55)/10 (45)	8 (73)/3 (27)	4 (36)/7 (64)	4 (36)/7 (64)	8 (80)/2 (20)
fever present, median (duration day)	4 [2–4]	4 [2–6]	3 [2–4]	4 [2–6]	3 [2–3]	3 [2–5]
virus type detected (n)	EV-D68 (6); HRV-C (4); HRV-A; RSV-B	RSV-B (5); EV-D68; HRV-A; HRV-B; HRV-C	ADV; HRV-B	RSV-A (9); CV-B4; EV-D68; HCoV-OC43; HRV-C	HRV-B (3); HCoV-OC43	HRV-A (3); HRV-C; HCoV-229E
**Symptoms**						
headache, n (%)	18 (90)	21 (95.5)	11 (100)	7 (63.5), 4 NA^‡^	11 (100)	10 (100)
cough, n (%)	18 (90)	22 (100)	11 (100)	11 (100)	8 (73)	5 (50)
malaise, n (%)	15 (75)	21 (95.5)	4 (36.5)	11 (100)	0	0
chills, n (%)	0	21 (95.5)	5 (45.5)	3 (27.5)	7 (63.5)	2 (20)
muscle ache n (%)	3 (15)	0	1 (9)	0, 4 NA	0	7 (70)
joint pain, n (%)	8 (40)	0	1 (9)	0, 6 NA	2 (18)	1 (10)
seizure, n (%)	0	0	0	1 (9)	0	0
sore throat, n (%)	12 (60)	4 (18)	11 (100)	3 (27.5), 8 NA	1 (9)	6 (60)
shortness of breath, n (%)	0	0	0	10 (91)	0	0
nausea, n (%)	0	0	0	9 (82)	0	0
vomiting, n (%)	1 (5)	0	10 (91)	8 (73)	0	0
abdominal cramps, n (%)	1 (5)	0	2 (18)	1 (9), 4 NA	0	0
Site Diagnosis*						
pharyngitis, n (%)	11 (55)	3 (13.5)	10 (91)	0	0	0
influenza, n (%)	7 (35)	5 (23)	1 (9)	0	0	7 (70)
pneumonia, n (%)	1 (5)	0	0	11 (100)	0	0
bronchitis, n (%)	0	1 (4.5)	0	0	0	0
upper respiratory infection, n (%)	0	13 (59)	0	0	0	0
malaria, n (%)	0	0	0	0	11 (100)	3 (30)
other, n (%)	1 typhoid fever	0	0	0	0	0

^‡^NA = Not available; HRV-A/B/C = human rhinovirus type A/ B/C; EV-D68 = enterovirus D68; HCoV = human coronavirus; ADV = adenovirus; RSV-A/B = respiratory syncytial virus A/B; CV-B4 = coxsackievirus B4.

*all samples tested negative for influenza virus, dengue virus and Plasmodium spp.in laboratory assays.

### KD02 cluster

**Figure 3:**
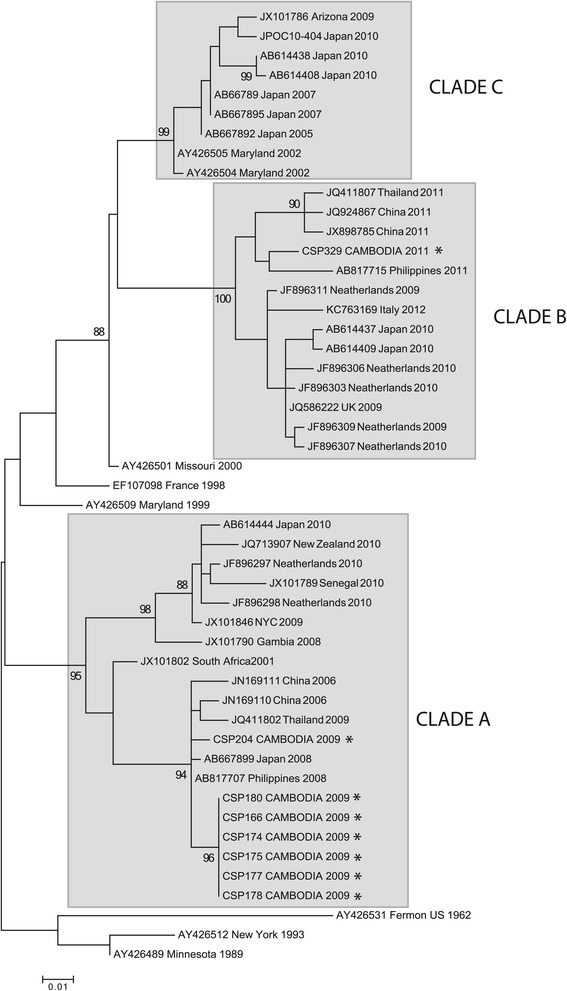
**Maximum-likelihood phylogenetic tree of EV-D68 based on a 339 base pair fragment of the VP1 gene.** For clarity, only select sequences were included in the tree. The three main clades are shown in gray. The sequences from the current study are indicated by *.

**Figure 4:**
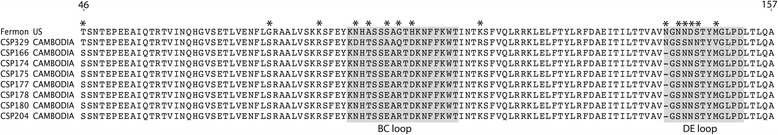
**Comparison of 112 amino acids of VP-1 from the EV-D68 Fermon strain to the eight Cambodian sequences generated in this study.** Coordinates are provided relative to the Fermon VP1 sequence (accession number AY426531). The putative antigen binding loops BC and DE are indicted in gray. The amino acid differences in the Cambodian strains relative to Fermon are indicated by *.

The KD02 cluster samples were collected from 20 patients in Kandal who presented for medical attention over an eight-day period in August, 2009. Eighteen had influenza-like illness (ILI), one had a presumptive diagnosis of typhoid fever and one was diagnosed with pneumonia (Table 1). Analysis of these samples by MassTag PCR detected a viral agent in 11 samples (Table 2). HEV was detected in six samples; one of them also contained RSV-B. To determine the HEV serotype, we amplified and sequenced a 600 nucleotide (nt) VP4-VP2 genome fragment from each sample. Sequence analysis indicated that all six HEV-positive samples contained a single strain of enterovirus 68 (EV-D68) (100% nt identity in VP4-VP2). Unlike most HEVs, EV-D68 is predominately a respiratory pathogen. Recent genetic analysis of EV-D68 indicated three main clades of the virus circulating worldwide [11]. To determine the clades circulating in Cambodia, we amplified and sequenced a 339 nt fragment of VP1 from all six samples. The Cambodian EV-D68 strain was most similar to EV-D68 strains originating from Asia between 2006 and 2010 (99% identity to strains from Japan, Philippines and China) and clustered with strains belonging to clade A, which has been implicated in recent EV-D68 outbreaks worldwide [11-14] (Figure 3). Comparison of amino acids within the VP1 relative to the EV-D68 Fermon reference strain revealed the majority of the substitutions were in the putative surface-exposed BC and DE loops (Figure 4). All six EV-D68 positive samples originated from adults (four men, two women) ranging between 24 to 71 years of age. Five of the six patients presented with pharyngitis, headache, cough and malaise. The exception, a 36-year-old male, presented with abdominal cramps, and joint pain, and was initially diagnosed with typhoid fever. Since both symptoms are atypical of EV-D68 infection, they may have been caused by a co-infecting agent.

**Table 2:** **Clinical description of virus positive cases in the KD02, KS02 and RK01 clusters**

**Site**	**Date enrolled**	**Virus detected**	**Age**	**Gender**	**Sore throat**	**Cough**	**Temperature**	**Site diagnosis**
KD02	25-Aug-2009	EV-D68	46	male	No	Yes	38.4	influenza
KD02	26-Aug-2009	EV-D68	71	female	Yes	Yes	38.4	pharyngitis
KD02	26-Aug-2009	EV-D68	36	male	No	No	38.8	typhoid fever
KD02	27-Aug-2009	EV-D68	24	female	Yes	Yes	38.3	pharyngitis
KD02	27-Aug-2009	EV-D68	38	male	No	Yes	38.7	pharyngitis
KD02	31-Aug-2009	EV-D68, RSV-B	51	male	Yes	Yes	38.7	pharyngitis
KD02	24-Aug-2009	HRV-C	4	male	No	Yes	39.0	pharyngitis
KD02	26-Aug-2009	HRV-C	11	female	Yes	Yes	38.6	pharyngitis
KD02	26-Aug-2009	HRV-A	59	female	Yes	Yes	38.5	pneumonia
KD02	26-Aug-2009	HRV-C	40	male	No	Yes	38.8	influenza
KD02	31-Aug-2009	HRV-C	37	male	Yes	Yes	39.2	pharyngitis
KS02	12-Oct-2009	RSV-B	8	male	No	Yes	39.0	influenza
KS02	13-Oct-2009	RSV-B	3	female	No	Yes	38.8	URI^^^
KS02	13-Oct-2009	RSV-B, HRV-C	3	female	No	Yes	38.5	URI
KS02	13-Oct-2009	RSV-B	12	male	Yes	Yes	39.5	pharyngitis
KS02	15-Oct-2009	HRV-A	2	female	No	Yes	39.0	URI
KS02	16-Oct-2009	EV-D68	60	female	No	Yes	38.6	influenza
KS02	16-Oct-2009	RSV-B	4	female	No	Yes	38.6	influenza
RK01	25-Jul-2011	RSV-A	3	female	Yes	Yes	38.5	pneumonia
RK01	1-Aug-2011	RSV-A	2	male	NA*	Yes	40.0	pneumonia
RK01	15-Aug-2011	RSV-A	5	female	NA	Yes	39.0	pneumonia
RK01	15-Aug-2011	RSV-A	3	male	NA	Yes	40.0	pneumonia
RK01	18-Aug-2011	CV-B4, RSV-A	2	male	NA	Yes	39.0	pneumonia
RK01	18-Aug-2011	EV-D68, RSV-A	4	male	NA	Yes	38.5	pneumonia
RK01	18-Aug-2011	HRV-C, RSV-A	4	male	NA	Yes	39.8	pneumonia
RK01	25-Aug-2011	HCOV-OC43, RSV-A	5	female	NA	Yes	38.5	pneumonia
RK01	12-Sep-2011	RSV-A	4	male	Yes	Yes	38.3	pneumonia

^^^URI: Upper Respiratory Infection; NA* = Data not available.

Five other samples from the cluster contained a HRV; amplification and sequencing of the VP4-VP2 genome fragment revealed that four of these were HRV-C and one was HRV-A (Table 2). Three HRV-positive samples originated from adults and two from children.

### KS02 cluster

The cluster of 22 cases from Kampong Speu province, collected within 7 days in October 2009, contained 16 patients <15 years of age. Upper respiratory tract infection was the most common diagnosis. 21 of 22 patients had complaints of headache (95%), malaise (95%), chills (95%), and all 22 had cough (100%) (Table 1).

Testing revealed that five cases were positive for RSV-B, all in patients 12 years old and under. One sample was also co-infected with HRV-C (Table 2). A single sample contained HRV-A. Of the six adult cases, one contained EV-D68. Sequence analysis indicates this virus (CSP-204) was similar to the strain from the Kandal cluster with 98.7% and 98.6% nt identity in the VP4-VP2 and VP1, respectively (100% amino acid (aa) identity in both proteins) (Figures 3 and 4).

### RK01 cluster

Between July 25^th^ and September 12^th^ of 2011 a cluster of 11 children less than five years of age from Ratanakiri received a diagnosis of pneumonia. Ten of them had shortness of breath; eight had nausea and vomiting. MassTag PCR detected RSV-A in nine cases (82%). Four RSV-A positive samples were co-infected with other viruses, identified as, coxsackie virus B4 (CV-B4), human coronavirus (hCoV-OC43), HRV-C, or EV-D68 (Table 1). Sequence analysis of a VP1 fragment amplified from this EV-D68 strain indicated that this strain was only 86% identical (92% aa identity) to the Cambodian strains from 2009, and clustered with EV-D68 sequences from clade B (Figures 3 and 4). The highest similarity was to strains reported from the Philippines in 2008 (99% nt identity in the VP1) [11,15,16].

### KD02 (2010), ST02, and KT02 clusters

The remaining three clusters comprising 32 cases all originated during the dry season in Cambodia (November through May). In cluster KD02, 10 of 11 patients were initially diagnosed with pharyngitis. All 11 patients in this cluster reported headache, cough and sore throat. ADV and HRV-B represented the lone viral agents detected in this cluster. In cluster ST02 all 11 cases were initially diagnosed with malaria and complained of fever (100%), cough (73%) and chills (63.5%). HRV-B was detected in three samples, and one sample contained CoV-OC43. In cluster KT02, 70% of the cases were diagnosed with influenza; the remaining 30% of cases were diagnosed with malaria (Table 1). HRV was detected in four of these samples (3 HRV-A and 1 HRV-C); CoV-229E was present in one sample.

## Discussion

Our analysis of 6 clusters of previously undiagnosed ARI from Cambodia revealed the presence of potential viral pathogens in 44% of cases. The agents most commonly detected were HRV, RSV, and HEV. Our results are similar to published data from a Cambodian children cohort study wherein the predominant agents found were HRV and RSV [17]; but differ in that we also report findings in adults wherein the predominant organisms found were HRV and EV-D68.

RSV is a frequent cause of respiratory disease in children and was the predominant agent detected in pediatric clusters in Ratanakiri (RK01; RSV-A) and Kampong Speu (KS02; RSV-B) respectively. The two clusters of RSV occurred during the rainy season suggesting that RSV infections may represent a significant cause of viral pneumonia in Cambodian children during this period [17-19].

EV-D68 was the primary agent detected in a cluster initially diagnosed as pharyngitis/influenza and was also detected in individual cases from two other clusters. Our study represents the initial report linking EV-D68 with respiratory disease in Cambodia. First isolated in the 1960s, EV-D68 is atypical among EVs in respiratory tract tropism [20,21]. Since the late 2000′s, there has been a global increase of respiratory disease outbreaks associated with EV-D68. EV-D68 has been reported in outbreaks of respiratory disease in Asia (China, Thailand, Japan, the Philippines), Europe (Italy, Finland, the Netherlands, the UK, France), Oceania (New Zealand), Africa (the Gambia, Senegal, and South Africa) and the United States [11-13,15,16,22-24]. The clinical presentation of EV-D68 infections in these outbreaks ranged from acute, mild illness to severe pneumonia, and, in rare instances, death. Retrospective studies have shown epidemic emergence of EV-D68 in some countries, leading to the hypothesis that evolution of the virus may have increased its fitness, enhancing its global expansion [11,13]. In our study we found that multiple clades of EV-D68 were circulating in Cambodia between 2009–2011, similar to reports from other countries [12,13,16]. We detected representatives of two distinct EV-D68 clades, both with variations in the putative antigenic sites relative to the EV-D68 reference strain. It has been suggested that changes within the antigenic sites of the capsid may have enabled the recent worldwide emergence of EV-D68 [25].

The majority of EV-D68 positive cases in our study originated from adults. Although EV-D68 was originally isolated from children, recent reports link EV-D68 to ARI in both children and adults, though the severity of symptoms may differ [13,26].

During the preparation of this manuscript, a large outbreak of respiratory disease in children linked to EV-D68 has occurred in the US [27]. This outbreak, which has resulted in large number of hospitalized cases, represents the first documented large-scale outbreak of disease due to EV-D68.

## Conclusions

Our findings demonstrate the utility of multiplex diagnostic assays for differential diagnosis of respiratory tract infections and underscore the increasing importance of EV-68 in respiratory disease.

## Materials and methods

### Sample collection

Beginning in December 2006, hospital-based surveillance of acute febrile illness was conducted in six provinces of eastern Cambodia: Kandal, Kampong Speu, Kratie, Ratanakiri, Stung Treng and Svay Rieng (Figure 1) [6]. Over a six-year period subjects were enrolled at 18 health clinics within these provinces. Ten were located in Kandal and Kampong Speu, approximately 40 to 75 Km from the capital Phnom Penh, respectively, whereas the others were located in more remote areas with difficult access during rainy seasons.

Between 2009 and 2012, 17,363 patients were enrolled and tested by PCR for influenza virus and dengue virus (upper respiratory swab (URS) and serum, respectively) and by Giemsa stain of whole blood for *Plasmodium* spp. Inclusion/Exclusion criteria, testing algorithm, specimen processing and testing were as described by Kasper *et al.* However the catchment area of the study changed as per Figure 1 [6]. Samples were collected under the auspices of study protocols #NAMRU2.2005.0004 and #037 NECHR approved by the U.S. Naval Medical Research Center Institutional Review Board and The Cambodian Ethical Committee for Health and Research, respectively. All research was in compliance with applicable federal regulations governing the protection of human subjects. All enrolled patients provided written consent. For patients <18 years of age, consent was obtained from a parent or legal guardian.

### Cluster definition

Patients enrolled at the same site within a two-week span with similar clinical symptoms were included in individual clusters. We selected five such case clusters for further analysis. Two clusters originated from Kandal, and one each from Kampong Speu, Steung Treng, and Kratie provinces (Figure 1). Additionally, we selected a cluster of children under five years of age who were diagnosed at the Ratanakiri (RK01) field site with pneumonia. Patients in this cluster were enrolled within a 50-day period. Together the six clusters represented 85 patients.

### MassTag PCR

Total nucleic acid was extracted from 250 μl of URS using the EasyMag automated extraction platform (BioMérieux). Total nucleic acid (TNA) was eluted in 40 μl. cDNA was generated using 10 μl of TNA and SuperScript II Reverse Transcriptase kit (Life Technologies). All samples were screened by MassTag PCR using respiratory panels composed of 15 known respiratory viruses [9,10]. The panels included primer sets for detection of 14 RNA viruses (influenza A and B, human respiratory syncytial virus *A* and *B* (RSV-A, RSV-B), human parainfluenza virus 1, 2, 3, 4A and 4B, human metapneumovirus, human coronavirus (hCoV) OC43 and 229E, human enterovirus (HEV)/rhinovirus (HRV)), and 1 DNA virus (adenovirus (ADV). All samples positive by MassTag PCR were confirmed by single agent PCR and dideoxy sequencing of the resulting amplification products.

### Data analysis

Mass Tag PCR data were analyzed using non-parametric statistics. The confidence score was calculated using fold inter-quartile range distance of the positive control or the test sample from the 95th percentile cut-point of the negative control distribution for a tag. The score is similar to parametric z-score but is distribution free.

### PCR and dideoxy sequencing

The list of primers can be found in Table 3. For each reaction, quantified positive control standards, samples, and non-template controls were used. In HEV-positive samples the serotype was identified using a consensus nested PCR assay that amplifies a 600-nucleotide region within the VP4-2 gene. The cycling conditions used for MassTag PCR were also used in individual PCR assays with the exception of the HEV VP4/2 nested assay (annealing temperature of 55°C).

**Table 3:** **Sequences of primers used in the study**

**FLUA-F**	**GTCTTCTAACCGAGGTCGAAACG**
FLUA-R	GCATTTTGGACAAAGCGTCTACG
FLUB-F	CACAGCAAAAACAATGAATGGA
FLUB-R	AGCACTTCCATTACATCCTTTGC
RSVA-F	AGATCAACTTCTGTCATCCAGCAA
RSVA-R	GCACATCATAATTAGGAGTATCAAT
RSVB-F	AAGATGCAAATCATAAATTCACAGGA
RSVB-R	TGATATCCAGCATCTTTAAGTATCTTTATAGTG
Coronavirus229E-F	GGCGCAAGAATTCAGAACCA
Coronavirus229E-R	TAAGAGCCGCAGCAACTGC
CoronavirusOC43-F	TGTGCCTATTGCACCAGGAGT
CoronavirusOC43-R	CCCGATCGACAATGTCAGC
HPIV1-F	TACTTTTGACACATTTAGTTCCAGGAG
HPIV1-R	CGGTACTTCTTTGACCAGGTATAATTG
HPIV2-F	GGACTTGGAACAAGATGGCCT
HPIV2-R	AGCATGAGAGCYTTTAATTTCTGGA
HPIV3-F	GCTTTCAGACAAGATGGAACAGTG
HPIV3-R	GCATKATTGACCCAATCTGATCC
HPIV4A-F	AACAGAAGGAAATGATGGTGGAAC
HPIV4A-R	TGCTGTGGATGTATGGGCAG
HPIV4B-F	AGAAGAAAACAACGATGAGACAAGG
HPIV4B-R	GTTTCCCTGGTTCACTCTCTTCA
EV/RV-F	TCCTCCGGCCCCTGAATGYGGCTAAT
EV/RV-R	GGAAACACGGWCACCCAAAGTA
MPV-F	GCGAGARATGGGYCCHGAATCTG
MPV-R	CCTGARGCATTDCCRAGAACAACAC
ADV-F	CCCMTTYAACCACCACCG
ADV-R	ACATCCTTBCKGAAGTTCCA
EV/RV VP4/2 round 1-F*	TCIGGIARYTTCCACCACCAICC
EV/RV VP4/2 round 1-R*	CTCCGGCCCCTGAATRYGGCTAA
EV/RV VP4/2 round 2-F*	ACCRASTACTTTGGGTGTCCGTG
EV/RV VP4/2 round 2-R*	CCGG YAAYTTCCASCACCA
EV-D68 VP1 round 1-F*	AACGCCGAACTTGGYGTG
EV-D68 VP1 round 1-R*	GGTAAGRGCACCAGTKGGT
EV-D68 VP1 round 2-F*	TCCCTAGCTTAAAYGCAGTTG
EV-D68 VP1 round 2-R*	CCAGTGGGTACRAACATTGC
Coronavirus-F*	GGTTGGGAYTAYCCTAARTGTGA
Coronavirus-R*	CCATCATCAGAWARAATCATCAT
RSVA-F*	GGTGCAGGGCAAGTGATGTTA
RSVA-R*	GCCAGCAGCATTGCCTAATAC
RSVB-F*	ATGGTTCAGGGCAAGTAATGCT
RSVB-R*	TCTCCTCCCAACTTCTGTGCA

* indicates primers used in individual PCR assays.

All EV-D68 sequences were deposited in GenBank under accession numbers KJ556320–KJ556335. All base pair coordinates are provided relative to the original 1962 EV-D68 isolate, the Fermon strain (GenBank accession no. AY426531). A Maximum-likelihood phylogenetic tree of EV-D68 based on a 339 nucleotide fragment of the VP1 gene was constructed using Mega 5.2 software.
